# Some Practical Observations in the Differential Diagnosis of Croup and Diphtheria

**Published:** 1876-11

**Authors:** William S. Stewart


### Some Practical Observations on the Differential Diagnosis and Treatment of Croup and Diphtheria.

By William S. Stewart, M. D.

Gentlemen:—In the early part of this session of interesting and instructive meeting of our Society, I had the honor of being present to hear our learned brother read a paper on the subject, "Are Croup and Diptheria Identical?" which you will find published in No. 213, vol. vi., of the *Medical Times*. It is well worth its place in all our libraries.

After listening to the discussion which followed, the incredulity expressed, and the effects produced upon the mind of others, perhaps, as well as on myself, I thought the subject, in relation to the general practitioner, too important to admit of any doubts, and that it deserves to be discussed and re-discussed until we are assured of the nature of both diseases, as we come in contact with them more or less every day.

I will not presume to review the ground gone over by my colleague in regard to the anatomical situations, the microscopic and chemical conditions, of the membranes formed; but rather trust in the evidence which we have when ushered into the chamber where all are anxiously intent on the announcement of the physician as to the nature of the disease that has already made an inroad in their happy home, and whieh, according to statistics, carry away so many of our dear ones.

In the discussion of this subject to which I have referred, the following language was used: "Probably only one out of twenty cases meet with and considered as croup would really be that affection. A physician would see twenty cases of diphtheria before he would see one case of true pseudo membranous laryngitis." If that is true, I fear there are many errors in the diagnosis of that disease.

I am disposed to think that there are as many cases of genuine croup to-day as there ever were. I am sure, however, that we are not called upon to treat all the cases that do occur, since almost every mother is able to recoguize it in its inception, and, being apprehensive of its invasion, is always prepared to combat it with suitable remedies.

Another evidence of this fact is, when sent for to see a case, instead of being requested to see a certain one who is sick, the messenger says, Come quickly, for Johnny, or Mary, or whoever it may be, has the croup, which implies not only that they are familiar with the disease, but have probably exhausted all the various domestic remedies for its arrest.

It is not a rare occurrence to be called in to see some individual in a family where, the night previous, perhaps, some other member of the same family has had an attack of croup and was treated successfully without any physician.

I think it very probable that the majority of cases of croup are so treated.

This can only be accounted for from the fact of the frequent occurrence of the disease, and the unseasonable hour to call in a physician, and the necessity for prompt measures being at once adopted.

I wish, before entering upon the discussion, to refer to another remark made in regard to diphtheria. That is, "I think there is more diphtheria in the minds of the physicians than in the throats of the patients."

I hope that remark is not applicable to any of the members of the regular profession, as it certainly reflects either incapacity or deception, neither of which ought to be tolerated among the conscientious and scientific, but both of which are almost necessary to the irregular and the quack.

It is practical knowledge that is needed, with a great deal of uncommon good sense, in order to arrive at distiction in any profession or calling; and especially is it the case in our profession, when we are regarded as the oracle whose decisions and directions are immutable, and the superiority of our remedies appearing of too high an order to descend even to our own familiar language. It seems to be a necessity that we resurrect the dead in order that we may minister to the living.

There seems to be a heavy cloud of mysticism which ought not to hang over the medical profession of to-day. I think the time has come when the intelligence of the community is sufficient to understand the what, the why, and the wherefore of our actions, and, consequently, could better appreciate the earnest efforts of the physician, and would not be so easily led astray by every new device that is placarded so as to allure the unsophisticated by its enticements. It is too true that in the allied profession there is much deception practised on the part of the doctor with his patients and friends, sometimes through ignorance and at other times by design, and from both causes, perhaps, are unwilling to give a disease its right name, but must magnify it so as to make the success of the treatment so astonishingly evident that its effects on the victim and his friends would be perfectly enchanting. Did you ever see such devotion as is exhibited by the converts to homœopathy? Is it not because "it is the God which they so ignorantly worship?"

In order, therefore, that the truth may be known, we must not only be educated ourselves, but we must be educators of those with whom we may be associated.

Before comparing these two diseases, I will first endeavor to define them separately.

Croup is inflammation of the trachea, which may extend up into the larynx or down into the bronchi. It occurs in young children after they are weaned, and is said to have a greater affinity for the male sex. The chief characteristic of croup is the formation of a membrane which lines the windpipe, the larynx, and bronchi as far as it extends. The gravity of the disease depends upon the obstruction produced by the membrane in respiration. The symptoms are generally ushered in after sunset or midnight; and is is presumable that the disease is produced by a sudden chilling of the secretions of the body.

The first indications of the disease are symptoms of catarrh, with sneezing, coughing, and horseness. It is the last symptom which is the signal for attention.

The cough is of a loud, ringing character, resembling somewhat the sound of a frog or the bark of a dog, and is so evident that one who has never heard it could not fail to recognize the symptom; this continues until the breathing becomes difficult and stridulous. If the disease were not promptly arrested, it would soon prove fatal to its victim. Other symptoms which accompany the disease are high inflammatory fever, flushed face, hot skin, thirst, frequent and hard pulse.

There is no morbid appearance in the pharynx nor difficulty in swallowing.

I will not stop here to describe true and false croup as distinct diseases, as both depend upon the same causes and have like symptoms. Suffice it to say that there are modifications in all diseases according to the constitutions which they attack, and the real difference is in degree rather than kind.

As to the symptoms of diphtheria.

The disease is ushered in with a chill, more or less fever, no cough, enlargement of the glands of the neck and throat; the patient complains of sore throat, difficulty in deglutition, pain and aching all through the system, indisposition from prostration. On examining the throat, we find white patches of membranous exudation deposited on the tonsils, it may be swollen flabulous uvula, the whole cavity of the upper jaw generally dotted over with fine drops of clear exudation. Sometimes we find a long membranous band extending down from the posterior nares, which is soft but very tenacious. As the disease progresses, the whole of the throat becomes involved. Not only does the uvula become covered, the roof of the mouth, the tonsils, the back part of the pharynx, but the disease extends up through the posterior nares until the whole passage-way of the nose is almost if not altogether closed.

The putridity of this membrane when left undisturbed is very offensive, and has a characteristic odor which, to a person of an acute organ of smell, can be recognized in entering the chamber.

I think it is, in my experience, as easily detected on first visit as the odor of a well-developed case of variola.

I wish particularly to call your attention to the fine drops of exudation, perhaps the size of a pin's head, which are frequently found dotting the upper and posterior part of the mouth, resembling somewhat drops of perspiration.

This is, to me, a certain indication of diphtheria. I have seen cases, where that condition was left undisturbed, and the deposit kept developing until the drops coalesced and united into one coagulated band that extended over the entire roof of the mouth with such evenness that it would evade your closest scrutiny were your attention not espeeially directed to it or you apprehensive of such a condition.

In contrasting the two diseases we find the following dissimilarity:

Croup is ushered in by a cough.
Diphtheria by a chill.
Croup is most frequent when there is greater humidity in the atmosphere and the east wind is prevailing.
Diphtheria does not depend upon meteorological changes.
Croup is not contagious.
Diphtheria most decidedly is.
Croup comes on suddenly.
Diphtheria may be tardy.
Croup is recognized by the croaking sound.
Diphtheria is known by the patches of membrane on the throat.
Croup must be promptly relieved.
Diphtheria is tardy in its resolution.
Croup does not affect the system.
Diphtheria is very prostrating.
Croup occurs most frequently in childhood and from two to five years.
Diphtheria occurs at all ages.
Croup is apt to occur very often in the same case.
Diphtheria may occur more than once in the same case, but the patient is not so liable to a second attack.

I might add also the contrast in the chemical condition of the urine and the blood, but, as that was referred to in Dr. Welch's paper, I will simply mention that the urine is not affected in croup, but in diphtheria it becomes albuminous. So also in the blood there may be an increase of normal constituent, fibrine, in croup, but in diphtheria there is a morbid condition of the blood not determined by increase of a normal constituent, and produced only by poison in the system. In consequence of the above conditions there are no offensive exhalations arising from croup, which are very manifest in diptheria.—*Phil. Med. Times.*

Owing to the length of the paper some interesting remarks on treatment are omitted.

